# Immune Thrombocytopenia Revealing Enriched IgG-4 Peri-Renal Rosai-Dorfman Disease Successfully Treated with Rituximab: A Case Report and Literature Review.

**DOI:** 10.3389/fmed.2021.678456

**Published:** 2021-06-16

**Authors:** Jerome Razanamahery, Sebastien Humbert, Jean-Francois Emile, Fleur Cohen-Aubart, Jean Fontan, Philippe Maksud, Sylvain Audia, Julien Haroche

**Affiliations:** ^1^Internal Medicine Department and Clinical Immunology, Dijon University Hospital, Dijon, France; ^2^Internal Medicine Department, Besancon University Hospital, Besançon, France; ^3^Department of Pathology, Ambroise-Paré Hospital, Assistance-Publique Hopitaux de Paris, Paris, France; ^4^Sorbonne Université, Assistance Publique Hôpitaux de Paris, Pitié-Salpêtrière Hospital, Internal Medicine Department 2, National Reference Center for Histiocytosis, Paris, France; ^5^Department of Haematology, Besancon University Hospital, Besançon, France; ^6^Department of Nuclear Medicine, Pitié-Salpêtrière Hospital, Paris, France

**Keywords:** immune thrombocytopenia, histiocytosis, Rosai Dorfman disease, Erdheim Chester disease, rituximab

## Abstract

Immune thrombocytopenia (ITP) is a rare autoimmune-mediated condition characterized by isolated thrombocytopenia (<100 G/L) after exclusion of other causes. Mostly primary, it is associated with hematological malignancy, autoimmune disorders, or infection in 20% of patients. It is exceptionally described in patients with histiocytosis, mostly in children (seven patients in literature). We report a case of a 69-year-old man with ITP leading to the diagnosis of histiocytosis. At ITP's diagnosis, the patient had elevated gamma-globulins leading to computed tomography showing bilateral peri-renal infiltration. The biopsy showed enriched IgG-4 peri-renal Rosai Dorfman disease with *MAP2K1* mutation, although peri-renal infiltration is highly suggestive of Erdheim-Chester disease. This overlapping association was described in men with mutation in *MAP2K1* gene. Macrophages are implicated in the pathophysiology of ITP in multiple ways, notably by the phagocytosis of opsonized platelets and their function of antigen-presenting cells able to stimulate autoreactive T cells. Histiocytic cells derivate from monocyte-macrophage lineage. Activation of macrophages in active histiocytosis is responsible for consequential platelet destruction in ITP associated histiocytosis. Finally, this case highlights a rare presentation of ITP revealing histiocytosis, both being efficiently treated with rituximab.

## Introduction

Immune thrombocytopenia (ITP) is a rare autoimmune disease characterized by isolated thrombocytopenia (<100 G/L) after exclusion of other causes (1). Although mostly primary, ITP is associated with chronic infections, connective tissue diseases, or hematological malignancies in 20% of patients (1). The association between histiocytosis and ITP is rare. It has been reported mostly in children (2–4); and in most cases, the diagnosis of both pathologies onset occurred several years apart.

Rosai-Dorfman disease (RDD) is a non-Langerhans cell histiocytosis characterized by histology demonstrating enlarged CD68+, CD1a−, and S100+ histiocytes with abundant lesions of emperipolesis (5). RDD locations are heterogeneous, but peri-nephric involvement is exceptional and usually consistent with Erdheim-Chester Disease (ECD). ECD is characterized by an iconic phenotype with long bone involvement, peri-nephric fat infiltration, and arteries' vascular sheathing (6). Histology usually demonstrates CD68+, CD1a−, and S100- histiocytes among fibrosis but is not as specific as in RDD. The first manifestations of both histiocytoses are heterogeneous but severe thrombocytopenia at diagnosis is uncommon.

Here, we report a case of ITP leading to RDD/ECD histiocytosis diagnosis successfully treated with Rituximab.

## Case Presentation

A 69-year-old man was referred for spontaneous epistaxis related to severe thrombocytopenia (7 G/L). He had a medical history of pancreatitis and took no medication. Physical examination showed isolated purpuric lesions of the lower limbs. Laboratory tests showed hemoglobin at 10.2 g/dL, white count cells at 5.36 G/L, platelets at 7 G/L without schistocytes on the blood smear. Coagulation tests were regular. Renal and liver functions were normal. Protein electrophoresis showed the protein level at 97 g/L (*N*: 60–80), albumin at 28.4 g/L (*N* > 40) and gamma globulins at 49.2 g/L (*N*: 8–13.5) without monoclonal gammopathy. Screening for infectious diseases (HIV/HBV/HCV) was negative. Immunological tests showed isolated antinuclear antibodies at 1/160 (*N*: <1/80) without antiphospholipid antibodies. Bone marrow aspiration showed no evidence for a myelodysplastic disorder nor for B9/B12 vitamin deficiency. The diagnosis of ITP was retained, and the patient received oral prednisone, 1 mg/kg/day for 4 weeks, resulting in clinical and biological response.

**Figure 1:**
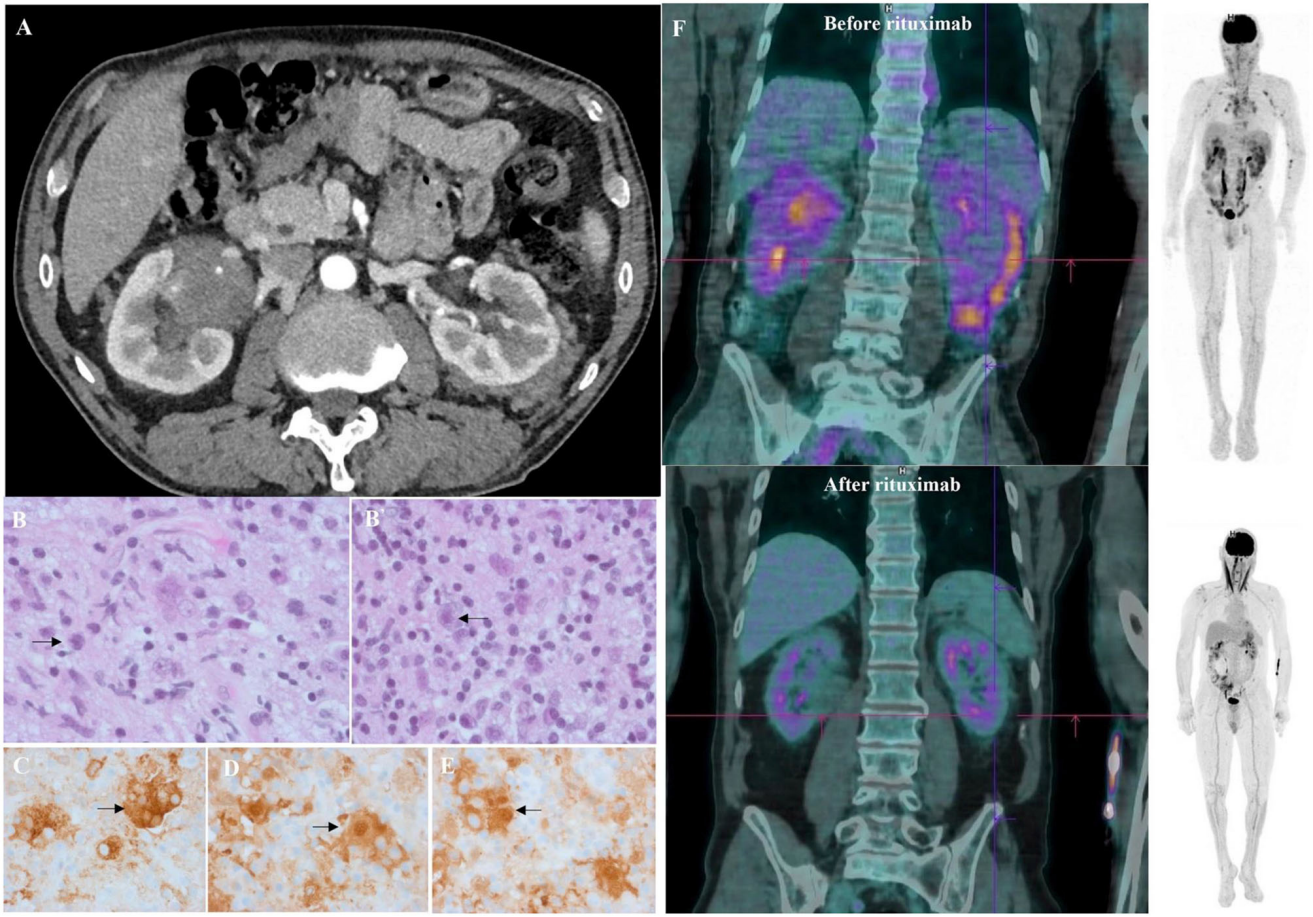
Clinical, radiological, and histological features of mixed Erdheim Chester Disease (ECD)/Rosai Dorfman Disease (RDD) histiocytosis. **(A)** Axial computed tomography (CT) of the patient demonstrating infiltration of peri-nephric fat defined as “hairy-kidney,” highly suggestive of ECD after 4 weeks of corticosteroids for ITP. **(B)** Tissue guided biopsy of perinephric lesions consistent with Rosai-Dorfman disease histology as shown by multinucleated histiocytes with large nuclei, abundant cytoplasm and lesions of emperipolesis (arrows). Haematoxylin and eosin staining (HES), original magnification ×400. Biopsy was performed 3 months after ITP diagnosis. **(B′)** Same samples showing perinephric lesions consistent with Rosai-Dorfman disease histology as shown by multinucleated histiocytes with large nuclei, abundant cytoplasm, and lesions of emperipolesis (arrows). Haematoxylin and eosin staining (HES), original magnification ×100. Biopsy was performed 3 months after ITP diagnosis. **(C)** Sample samples showing tissue infiltration with multinucleated histiocytes with CD163 expression on immunostaining (HES; immunohistochemistry, ×400) consistent with Rosai-Dorfman disease. **(D)** Same sample showing a strong expression of S100 protein (brown staining) by the multinucleated histiocytes (HES; immunochemistry, ×400) consistent with Rosai-Dorfman disease. **(E)** Same sample showing a strong expression of S100 protein (brown staining) by the multinucleated histiocytes (HES; immunochemistry, ×400) consistent with Rosai-Dorfman disease. **(F)** Sagittal ^18^FDG-PET CT-scan showing radiotracer uptake before (upper image) and after (lower image) rituximab infusions. An intense radiotracer uptake on “hairy-kidney” and a low tracer uptake on the testis was observed before treatment.

After the initial steroid course, the patient had a whole-body CT scan due to elevated gamma globulin level at ITP diagnosis. CT scan showed bilateral peri-nephric fat infiltration without other abnormalities (Figure 1A). At the biopsy time (3 months later), platelet count was 176 G/L, protein level at 86 g/L, albumin at 41 g/L, and gamma globulins at 23.1 g/L. The perinephric tissue biopsy showed infiltration with histiocytes, lymphocytes, and plasma cells rich in IgG-4 (130/high-power fields, IgG-4/IgG ratio: 25%; Figures 1B,B′). Histiocytes were CD68+, S100+, and CD1a-, with large nuclei and abundant lesions of emperipolesis (Figures 1C–E). Next-Generation Sequencing (NGS) on biopsy sample showed a c.395C>T variant on the *MAP2K1* gene. Although the histology was characteristic of enriched IgG-4 RDD, the bilateral peri-nephric fat infiltration was more consistent with ECD, but the bone scintigraphy was normal, and heart and brain MRIs showed no histiocytic locations. Because histiocytosis was non-symptomatic, no specific treatment was started. At 1-year follow-up, the patient experienced an ITP relapse with purpuric lesions of the lower limbs and a platelet count at 30 G/L. Protein electrophoresis showed the protein level at 114 g/L, albumin at 22 g/L, and gamma globulins at 62.6 g/L, with IgG1 level at 54.7 g/L (*N*: 3.8–9.3) and IgG-4 at 11.36 g/L (*N*: 0.039–0.864) without monoclonal gammopathy. Immunological tests showed isolated antinuclear antibodies at 1/320 and positive anticardiolipin IgG without clinical signs suggestive of lupus. Bone marrow aspiration was consistent with peripheral thrombocytopenia, although NGS detected somatic variants in several genes (*TET2, ASXL1, DNMT3A, JAK2*). ^18^F-fluorodeoxyglucose PET-CT showed intense radiotracer uptake in peri-nephric fat fibrosis, mediastinal lymph nodes, and a low tracer uptake on the testis (Figure 1F). The patient received a first infusion of rituximab 1,000 mg for chronic ITP. Ten days later, he experienced a new flare with epistaxis and a platelet count at 15 G/L without response to intravenous immunoglobulins (IVIg, 1 g/kg/day, day 1 and 2) and prednisone (1 mg/kg/day). Salvage therapy with dexamethasone 40 mg for 4 days and vinblastine 10 mg allowed a biological response. A second pulse of rituximab 1,000 mg was performed 15 days after. After a two-year follow-up, the patient is still on prednisone (5 mg/day) and considered in clinical, biological (platelet count at 286 G/L, IgG1 at 5.32 g/L, and IgG-4 at 0.033 g/L) and metabolic (Figure 1F) remission.

## Discussion

We report the first presentation of ITP leading to the diagnosis of histiocytosis. RDD location in peri-nephric space is rare (7), usually described as a solitary nodule or hilar mass or hypodense subcapsular infiltration. Bilateral peri-nephric fat infiltration is highly suggestive of ECD, raising the hypothesis of an overlapping histiocytosis, which was already described in men with testicular involvement and gain of function variant of *MAP2K1* gene (8). In this cohort, no patient had elevated IgG-4 plasmocytes on tissue biopsy or profound thrombocytopenia suggestive for ITP. The occurrence of somatic variants in bone marrow consecutive to clonal haematopoiesis of indeterminate potential (CHIP) is frequently reported in ECD patients (9), reinforcing the hypothesis of an “overlapping histiocytosis.”

Immunological conditions (i.e., autoimmune haemolytic anemia, IgG-4-related disease, systemic lupus erythematosus) have been reported in about 10% of patients with RDD (5, 10), even more frequently in a cohort of ECD patients (11). ITP has rarely been reported in patients with histiocytosis. In most cases, diagnosis of ITP and histiocytosis onset occurred several years apart (Table 1).

**Table 1:** Characteristics of previously reported patients with ITP and histiocytosis.

**References**	**Number of patients**	**Sex**	**Age at diagnosis of ITP**	**Age at diagnosis of histiocytosis**	**First symptoms of histiocytosis**	**Type of histiocytosis**	**Tissue disclosing histiocytosis**	**Mutation detected**	**Auto-immune features**	**Platelet count at diagnosis of histiocytosis**	**Specific treatment for ITP**	**Specific treatment for histiocytosis**	**Outcome (at publication date)**
Huang et al. (3)	1	M	70 years old	73 years old	Dyspnea, fatigue, systemic lymphadenopathy	RDD	Bone marrow	NA		23 G/L		Steroids	Dead
Lopetegui-Lia et al. (10)	1	M	52 years old	52 years old	Confusion	RDD	Sigmoid colon	NA	Antiphospholipid syndrome, pernicious anemia	69 G/L	Steroids^*^	Steroids^*^	Alive
Serra et al. (12)	1	M	39 years old	69 years old	NA	LCH	Bone marrow	NA		75 G/L	Steroids	none	Dead
Amorim et al. (13)	1	F	10 years old	10 years old	NA	LCH	Bones	NA		Between 30 and 70 G/L	Steroids/TPO agonist/Rituximab/Vincristine (during pregnancy)	Steroids/vinblastine/methotrexate	Alive
Lai and Pettit (4)	1	M	13 months	7 months	Enlarged mass of right temporal region	LCH	Skull	NA	AIHA	463 G/L	Steroids/IV-IG/TPO agonist (after chemotherapy)	Steroids/vinblastine/cladribine/cytarabine	Alive
Chen et al. (2)	1	F	10 years old	23 years old	Polyuria, polydipsia	LCH	Lymph node	NA		Over 100 G/L	Steroids	Cytarabine/zoledronic acid	Alive
Chen et al. (2)	1	M	22 months	11 months	Seborrheic lesions of scalp	LCH	Skin	NA	AIHA	Over 150 G/L	Ig-IV (after transplantation)	Vincristine/steroids/DAL-HX 83. Unrelated cordon blood transplantation.	Alive

*AIHA, Autoimmun hemolytic anemia; DAL-HX 83, Cyclophosphamide/doxorubicin/etoposide; LCH, Langerhans cell histiocytosis; RDD, Rosai-Dorfman disease; NA, not available*.

* *Concomitant treatment for ITP and histiocytosis*.

The pathophysiology of ITP is complex, macrophages playing a crucial role by phagocyting opsonized platelets in the spleen (14). Macrophages also participate in the stimulation of autoreactive T cells, among which T-follicular helper cells are essential for the stimulation and differentiation of autoreactive B-cell lymphocytes that produce antiplatelet antibodies (15).

A recent study highlighted an increased expression of M2-macrophage markers (CD163, CX3CR1) in the peripheral blood of ITP patients suggesting their potential immunomodulatory role in ITP pathogenesis (16).

Regarding histiocytosis ontogeny, cells originate from the fetal liver (17), the yolk sac (18), but mainly from the bone marrow (19, 20). Cells deriving from common bone marrow myeloid precursors give rise to blood monocytes who infiltrate several tissues and differentiate into dendritic cells and macrophages. Those monocytes can virtually infiltrate any tissues. Once in tissues, macrophages lose their migration ability (21) and become less sensitive to cell death signals (22). Those modifications are mandatory for tissue macrophage-homing and transformation into histiocytes (23).

In active histiocytosis, tissue macrophages from the same origins can be simultaneously activated and cause synchronous damages. Correlation between ITP and histiocytosis activity might be explained by macrophages activation into tissues resulting in platelet destruction.

The hypermetabolism of histiocytosis locations on PET-CT scan during ITP flare might support this postulate.

**Figure 2:**
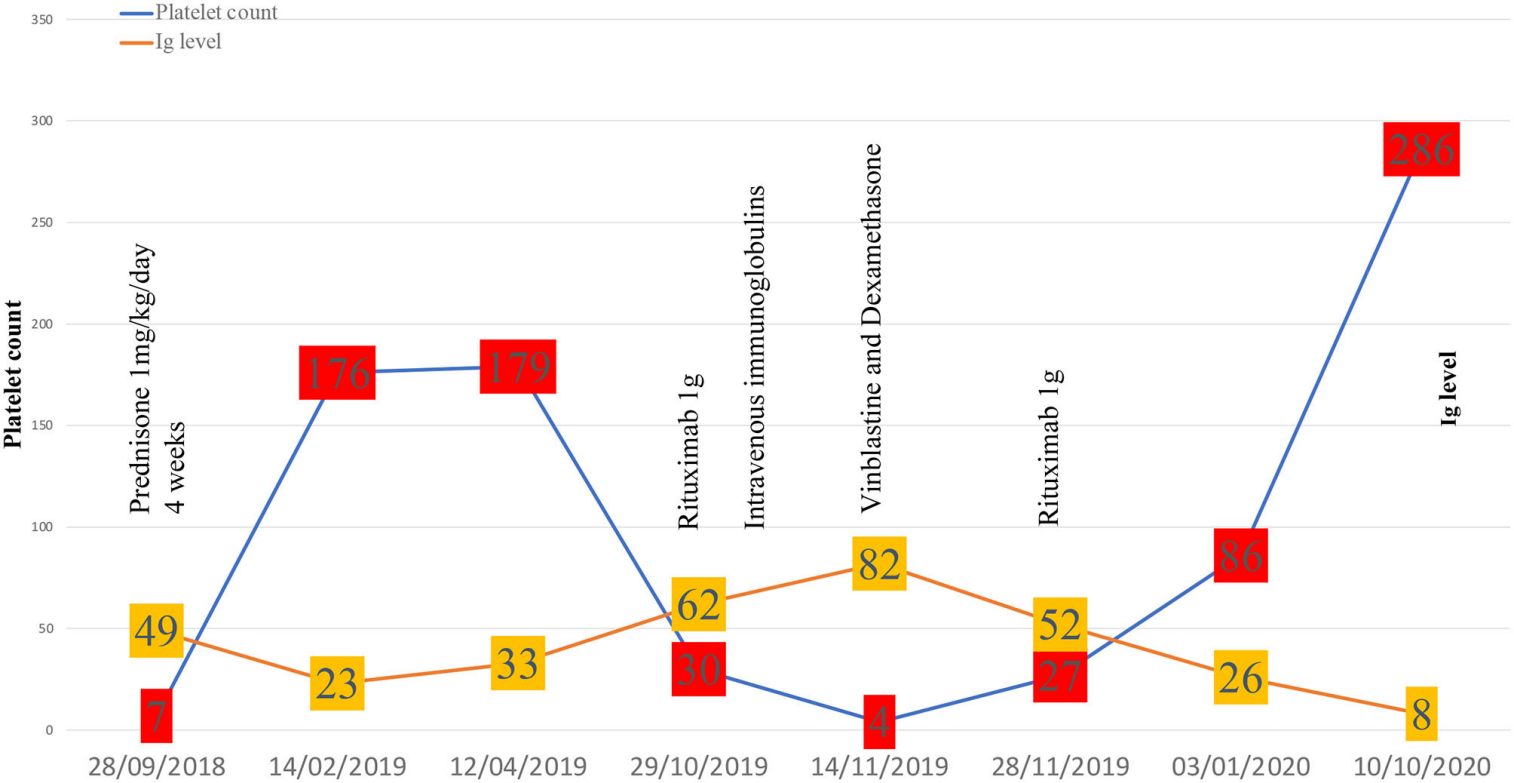
Evolution of immune thrombocytopenia.

Regarding treatments, steroids associated or not with IVIg depending on the haemorrhagic score, are the first-line therapy in newly diagnosed ITP. Second-line therapies such as thrombopoietin receptor agonists, rituximab are used in persistent ITP (>3 months) while splenectomy is dedicated to chronic ITP (>12 months). Salvage therapy with vinca-alkaloids has shown efficacy in refractory and life-threatening situations (1). In our case, rituximab was favored for persistent ITP because of its established efficiency in ITP (24), RDD (25), and IgG-4-related disease (26). Because of a relapse of ITP that was refractory to steroids and IVIg, vinblastine was proposed to rapidly increase platelet count before the response to rituximab was obtained. Finally, remission of both ITP and IgG-4-related features associated with RDD occurred 6 weeks after the last rituximab infusion. The complete therapeutic sequence has been reported in Figure 2.

In both pathologies, the long-term objective is to maintain remission out of treatment. Low-dose steroids are recommended for “auto-immune features” related to RDD (5, 25), but there is no consensus regarding maintenance therapy duration after remission. After 1 year of remission, steroids could be interrupted if the patient fulfills clinical, biological, and metabolic remission criteria. Surveillance with platelet count, Ig-G4 ratio at least every 6 months, and ^18^FDG-PET every year are useful to assess disease activity. In case of relapse of ITP, a new infusion of rituximab could be of interest if circulating B cells are detectable, as previously shown (27).

If not, treatment with azathioprine can be an interesting option for both ITP and RDD with efficacy reported in both pathologies (28, 29). Salvage therapy with dexamethasone and vinblastine could be a good option in severe bleeding. As MEK-inhibitors have shown a dramatic efficacy in histiocytic disorder (30, 31), Cobimetinib would be the best option for RDD life-threatening involvement. Interestingly, targeted therapies (BRAF and MEK-inhibitors) does not worsen auto-immunity in histiocytosis (11). Furthermore, severe thrombocytopenia has rarely been reported as a frequently associated side effect.

Although this association is not usual, ITP is more likely to be associated with histiocytosis rather than being primary, both being efficiently treated with rituximab.

## Data Availability Statement

The original contributions presented in the study are included in the article/supplementary material, further inquiries can be directed to the corresponding author/s.

## Ethics Statement

Written informed consent was obtained from the individual(s) for the publication of any potentially identifiable images or data included in this article.

## Author Contributions

JR collected the data and wrote the initial draft. J-FE confirmed histological features of RDD and determined the mutational status. All authors provided clinical, radiographic, histological data of the patient, participated in editing, and approved the final version of the manuscript.

## Conflict of Interest

FC-A and JH are investigators (FC-A being the PI) of an academic study on the efficacy of cobimetinib for treating histiocytoses. The remaining authors declare that the research was conducted in the absence of any commercial or financial relationships that could be construed as a potential conflict of interest.
